# An *xa5* Resistance Gene-Breaking Indian Strain of the Rice Bacterial Blight Pathogen *Xanthomonas oryzae* pv. oryzae Is Nearly Identical to a Thai Strain

**DOI:** 10.3389/fmicb.2020.579504

**Published:** 2020-10-21

**Authors:** Sara C. D. Carpenter, Prashant Mishra, Chandrika Ghoshal, Prasanta K. Dash, Li Wang, Samriti Midha, Gouri S. Laha, Jagjeet S. Lore, Wichai Kositratana, Nagendra K. Singh, Kuldeep Singh, Prabhu B. Patil, Ricardo Oliva, Sujin Patarapuwadol, Adam J. Bogdanove, Rhitu Rai

**Affiliations:** ^1^Plant Pathology and Plant-Microbe Biology Section, School of Integrative Plant Science, Cornell University, Ithaca, NY, United States; ^2^Plant Pathogen Interaction, ICAR-National Institute for Plant Biotechnology, New Delhi, India; ^3^Bacterial Genomics and Evolution Laboratory, CSIR-Institute of Microbial Technology, Chandigarh, India; ^4^Department of Plant Pathology, ICAR-Indian Institute of Rice Research, Hyderabad, India; ^5^Department of Plant Pathology, Punjab Agricultural University, Ludhiana, India; ^6^Department of Plant Pathology, Faculty of Agriculture at Kamphaeng Saen, Kasetsart University, Nakhon Pathom, Thailand; ^7^ICAR-National Bureau of Plant Genetic Resources, New Delhi, India; ^8^Rice Breeding Platform, International Rice Research Institute, Los Banos, Philippines

**Keywords:** bacterial blight of rice, single molecule real-time sequencing, transcription activator-like effectors, susceptibility genes, *SWEET* genes

## Abstract

The rice bacterial blight pathogen *Xanthomonas oryzae* pv. oryzae (*Xoo*) constrains production in major rice growing countries of Asia. *Xoo* injects transcription activator-like effectors (TALEs) that bind to and activate host “susceptibility” (*S*) genes that are important for disease. The bacterial blight resistance gene *xa*5, which reduces TALE activity generally, has been widely deployed. However, strains defeating *xa*5 have been reported in India and recently also in Thailand. We completely sequenced and compared the genomes of one such strain from each country and examined the encoded TALEs. The two genomes are nearly identical, including the TALE genes, and belong to a previously identified, highly clonal lineage. Each strain harbors a TALE known to activate the major *S* gene *SWEET11* strongly enough to be effective even when diminished by *xa*5. The findings suggest international migration of the *xa5*-compatible pathotype and highlight the utility of whole genome sequencing and TALE analysis for understanding and responding to breakdown of resistance.

## Introduction

*Xanthomonas oryzae* pv. oryzae (*Xoo*) causes bacterial blight of rice, a yield-reducing disease widespread in Asia and Africa (Nino-Liu et al., 2006). *Xoo* relies on type III secreted, transcription activator-like effectors (TALEs) that directly activate specific host genes, called “susceptibility” (*S*) genes, which contribute to disease development (Hutin et al., 2015). A TALE finds its DNA target by virtue of a central repeat region (CRR) in the protein composed of nearly identical, direct repeats of 33–35 amino acid residues. Residues at the twelfth and thirteenth positions in each repeat, together the “repeat-variable diresidue” (RVD), correspond to a single nucleotide in the effector binding element (EBE) in the DNA in a contiguous, code-like fashion such that the number and composition of RVDs predict the sequence of the EBE (Boch et al., 2009; Moscou and Bogdanove, 2009). The first residue of each RVD plays a stabilizing role and the second is the base-specifying residue. Characterized *Xoo* strains harbor 9 to nearly 20 different TALE-encoding (*tal*) genes, of which only one or two may encode a major virulence factor (Yang and White, 2004; Bogdanove et al., 2011; Hutin et al., 2015). All strains examined to date activate one of three members of clade III of the *SWEET* sucrose transporter gene family in rice (*SWEET11*, *SWEET13*, and *SWEET14*). These genes are major *S* genes and targeted by diverse TALEs from different strains (Hutin et al., 2015). In an experimental context, each of the other two members of *SWEET* clade III (*SWEET12* and *SWEET15*), and no other *SWEET* genes tested, also functioned as a major *S* gene (Antony et al., 2010; Streubel et al., 2013). *SWEET* activation apparently leads to sucrose export into the xylem vessels, facilitating *Xoo* proliferation and symptom development by an as yet uncharacterized mechanism.

Host resistance is the most effective means of controlling rice bacterial blight. To date, 46 bacterial blight resistance genes, called *Xa* (or *xa*) genes, have been identified from cultivated and wild rice species (Chen et al., 2020; Kumar et al., 2020; Neelam et al., 2020). The functions of most of the dozen or so *Xa* and *xa* genes that have been cloned and characterized relate to TALEs. A few are dominant, so-called executor resistance genes that function when transcriptionally activated by a TALE. Several are recessive, and all but one of these are alleles of a *SWEET* gene with a mutation at the EBE that prevents binding and activation by the cognate TALE, conferring resistance through reduced susceptibility. For example, *xa13* is a variant of *SWEET11* that lacks the EBE for TALE PthXo1 in its promoter and thereby confers resistance to strains that depend on PthXo1, such as the Philippines strain PXO99A (Yang and White, 2004; Chu et al., 2006). A strain can overcome *xa13* if it expresses a TALE (such as PthXo2, PthXo3, AvrXa7, or TalC) that activates an alternate clade III *SWEET* gene (Zhou et al., 2015). The recessive bacterial blight resistance gene that is not a *SWEET* allele, *xa5*, acts more broadly. It is an allele of the general transcription factor subunit gene *TFIIAγ5*. The protein encoded by the dominant allele is an apparent contact point between TALEs and the transcriptional machinery. The product of *xa5* harbors a single amino acid substitution that interferes with its interaction with TALEs and thereby reduces the ability of TALEs to activate their targets (Iyer-Pascuzzi et al., 2008; Huang et al., 2016). Notably, *xa5* is overcome by strains, like PXO99A, that carry PthXo1 (Huang et al., 2016). This “compatibility” was revealed to be due to the unusually strong activation of *SWEET11* by PthXo1, which even diminished in the *xa5* background is high enough to render the plant susceptible: strains with *SWEET* gene activators such as PthXo2, PthXo3, or AvrXa7, which activate their targets less strongly than PthXo1 activates *SWEET11* (Yang and White, 2004), are not able to cause disease in the *xa5* homozygous rice variety IRBB5. Furthermore, while those weaker *SWEET* gene activators expressed from a plasmid do not restore a *pthXo1* mutant of PXO99A to compatibility on IRBB5 plants, pthXo1 does render those *xa5*-incompatible strains compatible on IRBB5 plants (Huang et al., 2016).

The *xa5* gene has been widely deployed, both singly and in combination with other *Xa* genes (Jeung et al., 2006; Shanti et al., 2010; Khan et al., 2014). When deployed singly, like other *Xa* genes *xa5* has tended to break down over time (Khan et al., 2014). For example, in India, which is the second largest producer of rice behind China and has a highly diverse *Xoo* population (Midha et al., 2017), *xa5*-compatible *Xoo* isolates can be found throughout the country (Mishra et al., 2013; Yugander et al., 2017). In contrast, in Thailand, another major rice producer, *xa5* has largely remained effective (Wonglom et al., 2015); only recently have *xa5*-breaking Thai strains been isolated and they are not yet widespread (Wonglom et al., 2015). We chose an *xa5*-compatible strain from India, IX-280, isolated in Andhra Pradesh (Yugander et al., 2017) and one from Thailand, SK2-3, isolated in Sukhothai Province (Wonglom et al., 2015; Supplementary Figure S1) for genome sequencing to gain insights into their ability to overcome the resistance gene. We report here a comparison of the genomes of these two strains, with a focus on their TALE content. The results reveal surprising, near perfect identity of the two genomes, suggesting international migration, and a TALE repertoire that explains compatibility with *xa*5.

## Materials and Methods

The authors state that the experimental work with *Xanthomonas oryzae* was conducted in accordance with pertinent regulatory policies.

### Genomic DNA Extraction and Sequencing

DNA for complete-genome sequencing was isolated using the protocol described by Booher et al. (2015) with the following two modifications: after overnight culture and centrifugation, extracellular polysaccharide was removed by washing the bacterial pellet 7–8 times with NE buffer (0.15 M NaCl and 50 mM EDTA), and after cell lysis, DNA was extracted four times with phenol/chloroform and once with chloroform/isoamyl alcohol. For each strain, 4–7 μg of genomic DNA was used to prepare a 20 kb library and each library was sequenced by SMRT technology to >150X genome coverage using P6-C4 chemistry (Pacific Biosciences, Menlo Park, CA, USA), as described (Booher et al., 2015).

### Genome Sequence Assembly

*De novo* assembly of the sequence reads was performed using HGAP v.2.0 (HGAP2) and HGAP v. 3.0 (HGAP3) (Chin et al., 2013) as described (Booher et al., 2015). Since TALE encoding (*tal*) genes are often clustered and their repetitive sequences can lead to misassembly even using long-read technology, *tal* gene-containing regions were separately assembled using the PBX toolkit, a pipeline that uses long, *tal* gene sequence-containing seed reads to assemble *tal* clusters with more accuracy (Booher et al., 2015). Length cut-off settings used for these seed reads were 16 kb (pbx16000), 12 kb (pbx12000), or 10 kb (pbx10000). After HGAP and PBX assemblies were completed, the HGAP assemblies with the fewest unitigs and the majority of the *tal* gene sequences found by PBX were chosen for manual closure and finishing.

### Genome Finishing, Assembly Verification, and Annotation

To finish the genomes, the circular assemblies were polished twice more with Quiver and then checked for structural variants and misassemblies using PBHoney (English et al., 2014). The *tal* gene repertoires were verified by consensus with the local *tal* assemblies made with PBX and by Southern blots of genomic DNA digested with either *Bam*HI or *Sph*I, or with *Bam*HI and *Eco*RI, and probed with the *tal* gene specific probe pZWavrXa7 (Yang and White, 2004). To confirm the absence of plasmids smaller than 20 kb that could have been excluded during library preparation, total DNA was prepared and examined by agarose gel electrophoresis as described, using *Xanthomonas campestris* pv. vesicatoria 85-10, which has four plasmids, as a positive control (Booher et al., 2015). After finishing and assembly verification, genomes were annotated using the NCBI Prokaryotic Genome Annotation Pipeline (Tatusova et al., 2016), and *tal* gene annotations were manually corrected.

### Genomic Comparisons

For structural comparison, complete genomes were aligned using progressiveMauve (Darling et al., 2010) in the MegAlign Pro module of the DNAStar Suite (Lasergene 13.0.0.357) with default settings. For phylogenetic analysis, complete and draft genomes were aligned using Mauve v2.3.1 (Darling et al., 2004), and core alignment was used to infer phylogeny using PhyML v3.1 (Guindon et al., 2010). The core alignment and maximum likelihood tree were further subjected to ClonalFrameML (Didelot and Wilson, 2015) analysis with 100 bootstrap replicates to refine the phylogeny considering the impact of recombination. The ClonalFrameML tree was visualized using iTOL v3 (Letunic and Bork, 2016).

### TALE Analysis

All *tal* gene sequences were extracted using the PBX exporter (Booher et al., 2015) or AnnoTALE (Grau et al., 2016). Orthology of IX-280 and SK2-3 TALEs to previously sequenced TALEs was determined using FuncTAL (Perez-Quintero et al., 2015) and AnnoTALE (Grau et al., 2016). RVD or amino acid sequence was used as input for FuncTAL and DNA sequence for AnnoTALE. AnnoTALE class builder files used to assign TALEs to families were downloaded on July 1, 2017. The results from the two tools were consistent.

### Bacterial and Plant Growth Conditions and Disease and Gene Expression Assays

Plants were grown in a growth chamber maintained at 28°C and 85% relative humidity with a photoperiod of 12 h. *Xanthomonas* strains were cultured at 28°C on modified Wakimoto agar or glucose yeast extract medium. For the disease assay, bacterial cells were resuspended in sterile water at an OD_600_ of 0.5 and clip-inoculated (Kauffman, 1973) to fully expanded leaves of 40–45-day-old plants. Lesions were measured 14 days later. For gene expression assays, bacterial suspensions at an OD_600_ of 0.2 were infiltrated into leaves of 3-week-old plants using a needleless syringe. Water was used for mock inoculation as a control. The inoculated portions of leaves were harvested 24 h later, and total RNA was extracted using the PureLink™ RNA Mini kit (Invitrogen, Carlsbad, California, USA) following the manufacturer’s instructions. RNA was further treated with DNase (Invitrogen) to remove genomic DNA contamination. Quality and quantity of RNA were analyzed by 1.0% agarose gel electrophoresis and spectrophotometry using a Nanodrop (Thermo Scientific, Waltham, Massachusetts, USA). cDNA was generated from 1 μg purified RNA using the Superscript™ Vilo™ cDNA synthesis kit (Invitrogen) with random primers. Quantitative real-time PCR (qPCR) was performed on a Light cycler® 480 Instrument II (Roche Molecular Diagnostics, Santa Barbara, California, USA). About 250 ng of cDNA was used for each qPCR reaction with gene specific primers (Supplementary Table S1). Each gene was tested with three biological replicates, with three technical replicates each. The average threshold cycle (Ct) was used to determine the fold change of gene expression. The expression of each gene was normalized to the expression of the 18S rRNA gene. The 2^-ΔΔCt^ method was used for relative quantification (Livak and Schmittgen, 2001).

## Results

### Assembly of the Complete IX-280 and SK2-3 Genomes

Single molecule real-time (SMRT) DNA sequence data for IX-280 assembled using either HGAP2 or HGAP3 (see Methods) resulted into two contigs, corresponding to a chromosome and 43 kb plasmid. We named the plasmid pXOO43. The HGAP2 assembly, though it yielded an intact, self-complementary chromosomal contig, collapsed one cluster of four *tal* genes into three, indicated by a coverage spike in that cluster. A comparison of the ends of the misassembled cluster to pbx12000 and pbx16000 assemblies generated using the PBX toolkit (Booher et al., 2015) showed overlap with several that included an intact cluster of four *tal* genes. We chose a pbx16000 contig assembled using settings of 3,000 kb read overlap and 97% read identity to replace the misassembled cluster in the HGAP2 assembly. We also verified the presence of the cluster of four *tal* genes in the raw sequence of IX-280. To further confirm our final assembly, we obtained additional long reads from a separate DNA preparation of the same isolate and reassembled with HGAP3 using all available reads; the resulting HGAP3 assembly was consistent with the manually corrected HGAP2 assembly.

HGAP2 and HGAP3 assemblies of SK2-3 yielded a single chromosomal contig, but each terminated at a partial cluster of four *tal* genes. The intact cluster was present in pbx10000 assemblies. We selected a contig assembled using settings of 3,000 kb read overlap and 97% read identity to replace the broken cluster in the HGAP2 assembly and manually closed the genome.

The quality-control tool PBHoney (English et al., 2014) indicated no major inversions, deletions, or duplications in the assemblies. The proportion of mapped reads to post-filtered reads was 94.9% for IX-280 and 92% for SK2-3. Coverage graphs for the final assemblies showed no unusual peaks or dips that might indicate collapsed or expanded genomic repeats. PBX results were consistent with *tal* gene sequences extracted from the genomes, as were Southern blots hybridized with a *tal* gene-specific probe (Supplementary Figure S2). Separate DNA extraction and gel electrophoresis for both strains confirmed the absence of any small plasmids that might have been missed by SMRT sequencing (not shown).

### Comparison of the IX-280 and SK2-3 Genomes

The IX-280 plasmid pXOO43 has not been found in other *Xanthomonas* genomes, but some regions have a high degree of nucleotide identity with regions of pXAC64 from *Xanthomonas citri* ssp. citri (da Silva et al., 2002). There are no predicted type III effector genes on the plasmid, but it harbors a cluster of genes annotated as type VI secretion genes. Associated with this cluster is an apparent operon containing *pemK*, encoding a toxin in a toxin/antitoxin system (Agarwal et al., 2007), and a gene encoding a protein of the XF1863 family, hypothesized to function as its antitoxin (Makarova et al., 2009). None of the pXOO43 content is found in the SK2-3 genome.

**Figure 1:**
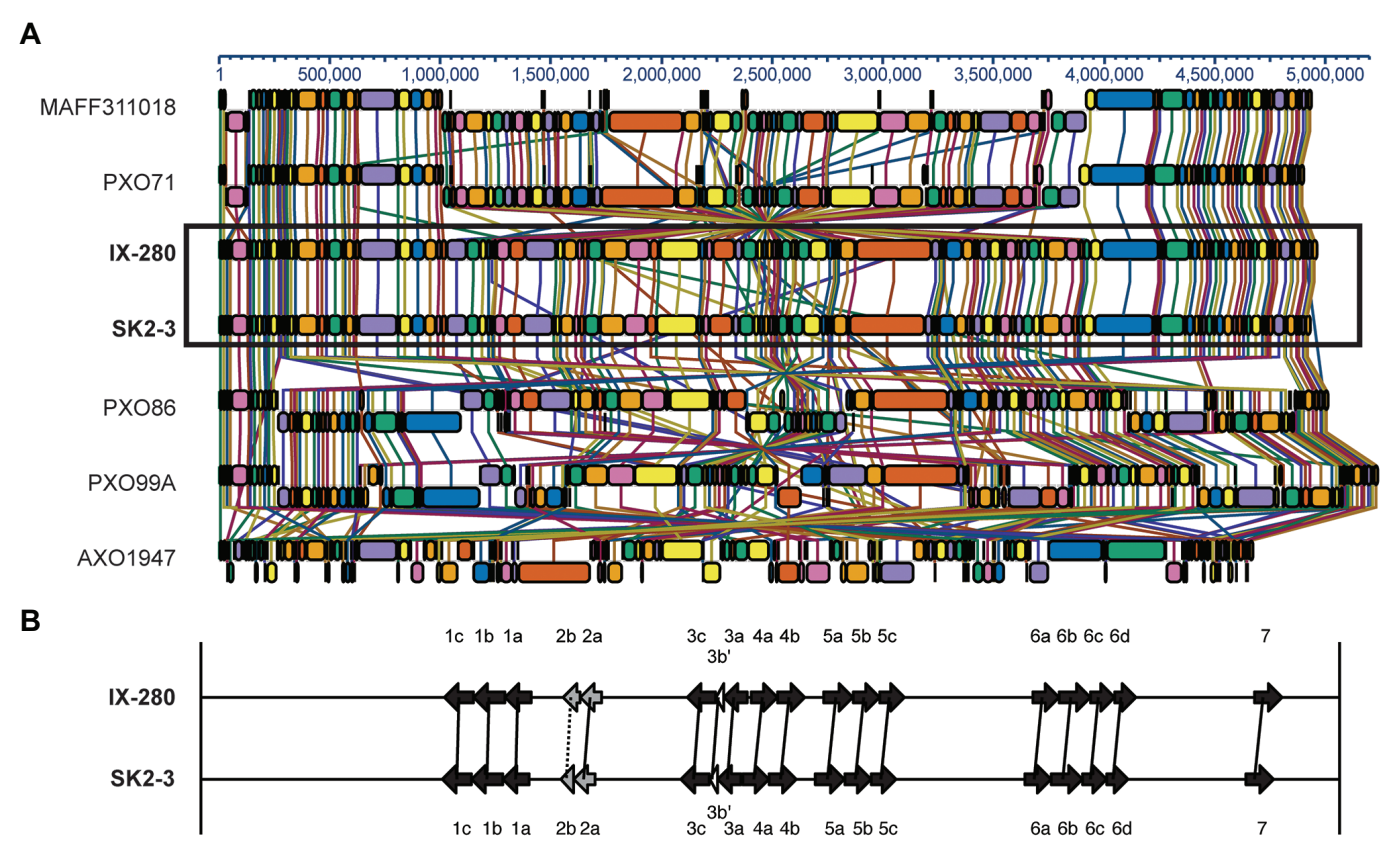
Synteny between IX-280 and SK2-3 genomes and comparison of their *tal* genes. **(A)** Progressive Mauve alignment of the chromosomes of IX-280 and SK2-3 and other representative *Xoo* strains. **(B)** Map of the *tal* genes in IX-280 and SK2-3. Black arrows represent full-length *tal* genes, gray arrows truncTALE genes, and white arrows *tal* pseudogenes. Solid lines connect *tal* genes with >99% nucleotide identity and identical RVD sequence, and dotted lines connect less similar but clearly orthologous genes.

The IX-280 and SK2-3 chromosomes are entirely syntenous (Figure 1A and Supplementary Figure S3), including the *tal* genes, which show no duplications, deletions, or rearrangements in one genome relative to the other (Figure 1B). To determine how the genome structure of IX-280 and SK2-3 compares with that of other *Xoo* strains, we aligned the genomes with those of select other strains representing three East Asian lineages and a more distant African lineage (Quibod et al., 2016): Philippines strain PXO71 and Japanese strain MAFF311018 representing lineage PX-A, Philippines strain PXO86 representing lineage PX-B, Philippines strain PXO99A representing lineage PX-C, and the African strain AXO1947. The alignment shows no relationship between geographic area of isolation and genome arrangement (Figure 1A). Like IX-280 and SK2-3, the genome structures of PXO71 (Philippines) and MAFF311018 (Japan) are similar to one another, despite the strains being from different countries. In contrast, PXO86, PXO71, and PXO99A, all from the Philippines, have undergone genomic rearrangements relative to one another. The genome structure of the African strain, AXO1947, is distinct from those of the other *Xoo* strains, showing some of the genomic variability encompassed by the species. Though there are areas of similarity, the genomic arrangement of IX-280 and SK2-3 is not shared by any of the other strains.

### IX-280 and SK2-3 Belong to a Highly Clonal Lineage

**Figure 2:**
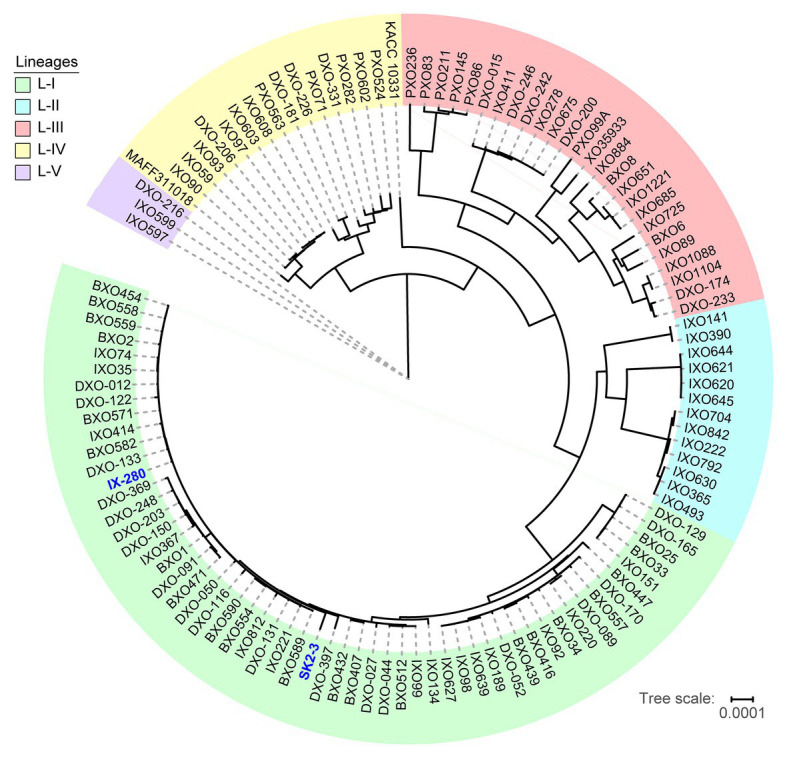
Positions of IX-280 and SK2-3 on a clonal lineage tree derived from genomic sequences of 100 Indian *Xoo* strains and other *Xoo* strains from Asia. Lineages are block shaded in different colors. IX-280 and SK2-3 (blue font) are in lineage L-I.

The striking genomic similarity of IX-280 and SK2-3 despite their geographic separation led us to explore their relatedness with other *Xoo* strains more broadly. Using draft (short-read derived) genome sequences of 100 Indian *Xoo* strains previously subjected to phylogenetic analysis (Midha et al., 2017) as well as several complete Asian *Xoo* genomes, we generated a phylogenetic tree using regions not affected by recombination. The previous phylogenetic analysis of the 100 Indian strains had revealed five lineages (Midha et al., 2017). Both IX-280 and SK2-3 map to the youngest and a highly clonal lineage, L-I (Figure 2). Of the strains examined, SK2-3 is the only non-Indian strain in this lineage.

### The TALE Repertoires Are Nearly Identical and Include a PthXo1 Ortholog

**Figure 3:**
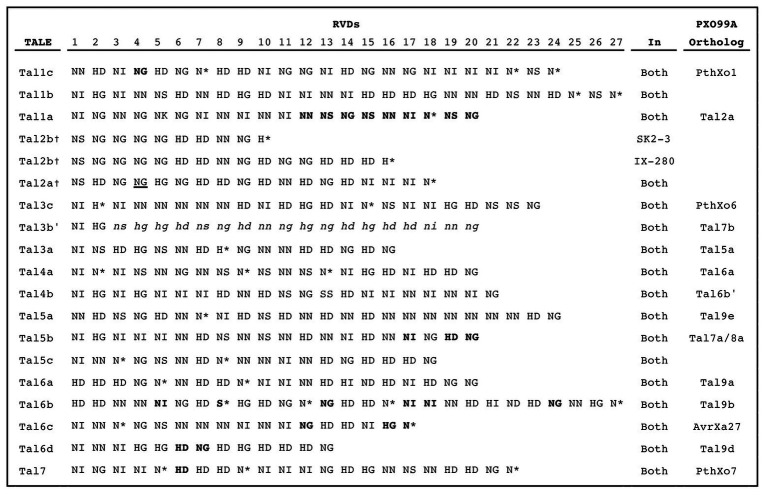
RVD sequences of IX-280 and SK2-3 TALEs. RVDs in bold are different in PXO99A orthologs. A dagger indicates a truncTALE. The underlined RVD of Tal2a resides in a truncated (28 aa) repeat. Lower case italicized RVDs are untranslated following a frameshift. An asterisk indicates that the second amino acid in the RVD is absent, resulting in a 33 aa repeat.

The TALE repertoires of IX-280 and SK2-3 each consist of 15 TALEs and two truncTALEs, which are TALE variants with shortened N- and C-termini that can function as suppressors of resistance mediated by certain non-executor resistance genes (Ji et al., 2016; Read et al., 2016); each strain also harbors a *tal* pseudogene (Figure 3). The RVD sequence of each IX-280 TALE and truncTALE is identical to that of its counterpart in SK2-3, except for the truncTALE Tal2b, of which repeats 10–15 are missing in SK2-3 (Supplementary Figure S4). Since truncTALEs do not bind DNA and a specific RVD sequence is not critical to their function (Read et al., 2016), this difference in Tal2b between the two strains is likely functionally irrelevant.

Tal1c of both strains is an ortholog of PthXo1 (Figure 3), which likely explains the ability of each strain to overcome *xa5*. PthXo1 in IX-280 and SK2-3 differs from PthXo1 in PXO99A at one RVD, but the base-specifying residue of that RVD is the same (Supplementary Figure S4). The strains harbor no other TALE predicted to target the promoter of any clade III *SWEET* gene. Notably, a nearly identical ortholog of PthXo7, the PXO99A TALE that induces *TFIIAγ1*, is also present in both strains (Tal7). Compatibility with *xa5* had been postulated to be due to activation of the paralog *TFIIAγ1* by PthXo7 (Sugio et al., 2007), but it was recently shown that only TFIIAγ5, and not TFIIAγ1, interacts *in planta* with tested TALEs (Yuan et al., 2016).

**Figure 4:**
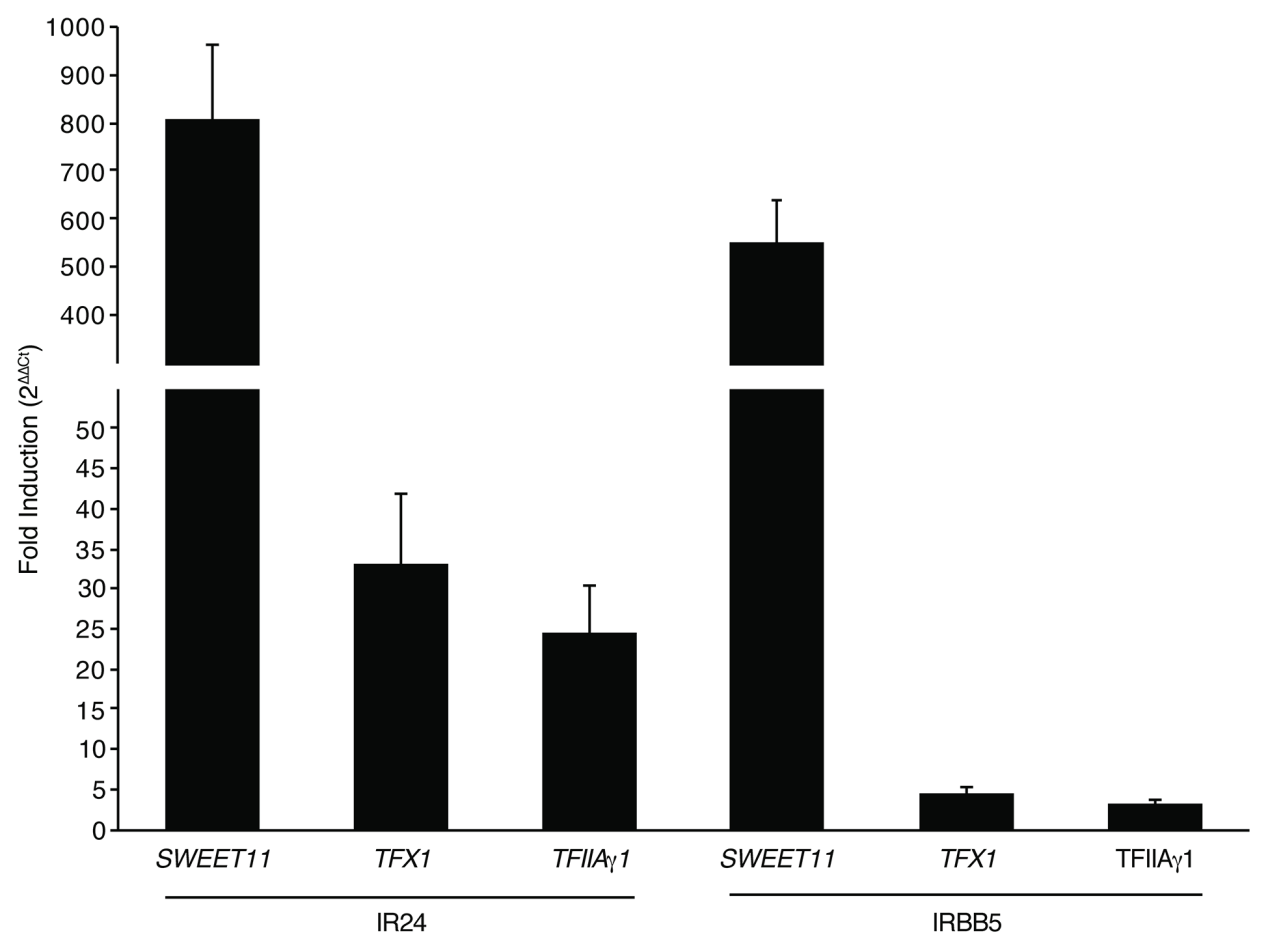
Induction of *SWEET11*, *TFX1*, and *TFIIAγ1* by IX-280 in IR24 vs. IRBB5 plants. Shown is fold induction in IR24 (*Xa5*) and IRBB5 (*xa5*) at 24–27 h after inoculation by syringe infiltration of IX-280 relative to mock (water)-inoculated leaves, measured by qRT-PCR. Each bar represents the mean of three replicates. Error bars represent standard deviation.

Based on the presence of the PthXo1 ortholog Tal1c, we hypothesized that IX-280 induces *SWEET11* sufficiently for virulence in IRBB5 plants. We compared expression of *SWEET11* in plants of the near-isogenic line IR24, which carries the dominant (non-functional with respect to resistance) allele, *Xa5*, and in IRBB5 plants, inoculated with IX-280, relative to mock inoculated plants, using quantitative RT-PCR of RNA harvested at 24 h. *SWEET11* was induced 799 fold in IR24 and 553 fold in IRBB5 (Figure 4). IX-280 and SK2-3 harbor an ortholog of the PXO99A TALE PthXo6, Tal3c, in addition to the PthXo7 ortholog, Tal7. PthXo6 induces the bZIP transcription factor gene *TFX1*. Thus, for reference, we also examined expression of *TFX1* and *TFIIAγ*1. Each of the transcription factor genes was moderately induced (20–35-fold) in IX-280-inoculated IR24 leaves relative to mock (Figure 4). This induction provides evidence that Tal7 and Tal3c are delivered and functional, and that the single RVD difference between PthXo7 and Tal7 does not impact targeting of *TFIIAγ1*. In IRBB5, *TFX1* and *TFIIAγ*1 induction was reduced to just 3–5-fold relative to mock (Figure 4). The results are consistent with the observation that the *xa5* allele reduces generally the ability of TALEs to induce their targets (Yuan et al., 2016) and suggest that, like PthXo1, Tal1c activates *SWEET11* strongly enough to enable IX-280 and SK2-3 to overcome *xa5*.

## Discussion

In this study, we determined that an *xa5* resistance-breaking strain of *Xoo* from India is nearly identical to one from Thailand. We further determined that the strains harbor a TALE known to activate the major *S* gene *SWEET11* strongly enough to be effective even when diminished by *xa*5. The genome comparisons we carried out (Figure 1A) and comparisons published elsewhere (Salzberg et al., 2008; Quibod et al., 2016) demonstrate the high level of variability in genome structure across different strains of *Xoo* and a general lack of relationship between genome structure and the geographical location at which a strain was isolated. Like other *Xoo* strains, both IX-280 and SK2-3 contain hundreds of IS elements and other transposons in their genomes (Table 1) that likely contribute to genome plasticity (Salzberg et al., 2008; Booher et al., 2015). Despite the overall genome structure variability in the species and the geographic separation of IX-280 and SK2-3, strikingly these two strains are part of a young and highly clonal lineage prevalent in India, L-I (Midha et al., 2017), in which no other characterized, non-Indian strains we examined clustered. This observation and the relative rarity of *xa*5 compatibility in Thailand (Wonglom et al., 2015) suggest introduction of SK2-3 or a recent progenitor from lineage L-I to Thailand directly, or indirectly, from India. Since we cannot rule out L-I having originated outside of India, however, it is alternatively possible that members of the lineage were introduced separately to Thailand and to India.

**Table 1:** The IX-280 and SK2-3 genome assemblies.

	IX-280	SK2-3
Chromosome	4,963,593 bp	4,934,446 bp
Plasmid	42,975 bp	-
Final coverage	164.0x	156.4x
% Mapped reads	94.6%	92.0%
Annotated genes	5,041	4,926
Annotated IS elements	411	407
Annotated transposases	730	698
*tal* genes	17	17

The basis for the compatibility of IX-280 and SK2-3 with *xa5* is almost certainly their PthXo1 ortholog, Tal1c. The single difference in RVD sequence between Tal1c and PthXo1 does not affect the base specifying residue (Supplementary Figure S3), so the two proteins can be expected to function the same; and IX-280, like PXO99A, is able to strongly activate *SWEET11* even under the dampening effect of *xa5* (Figure 4). Induction of *TFX1* by Tal3c (the PthXo6 ortholog) and of *TFIIAγ1* by Tal7 (ortholog of PthXo7), though reduced by *xa5*, may also contribute. PthXo6 is a demonstrated virulence factor and *TFX1* its verified *S* gene target (Sugio et al., 2007). And, studies suggest that activation of *TFIIAγ1* by PthXo7 contributes to susceptibility. Heterologous expression of PthXo7 in the *xa5*-incompatible strain PXO86 rendered the strain weakly virulent on IRBB5 plants (Sugio et al., 2007), and silencing of *TFIIAγ1* decreased susceptibility to PXO99A, even in an *Xa5* background (Yuan et al., 2016). We observed that despite induction of *TFIIAγ*1 by Tal7, activation of *SWEET11*, *TFX1*, and of *TFIIAγ*1 itself remain dampened in IRBB5 relative to IR24 (Figure 4). Thus, activation of *TFIIAγ1* by Tal7 appears to contribute to susceptibility in some way other than providing a substitute for *TFIIAγ5*.

The clonality of IX-280 and SK2-3 indicates that immigration contributes to evolution of local *Xoo* populations. The discovery of a PthXo1 ortholog in these strains highlights the utility of complete genome sequence- and TALE analysis-based monitoring to understand breakdown of resistance genes. The results also highlight the need to continue to develop local varieties with different individual, or better, stacked resistance genes, for rapid deployment.

## Data Availability Statement

The datasets presented in this study can be found in online repositories. The names of the repository/repositories and accession number(s) can be found at: https://www.ncbi.nlm.nih.gov/genbank/, CP019226

https://www.ncbi.nlm.nih.gov/genbank/, CP019227

https://www.ncbi.nlm.nih.gov/genbank/, CP019515

https://www.ncbi.nlm.nih.gov/genbank/, SRR5989134

https://www.ncbi.nlm.nih.gov/genbank/, SRR5990719

https://www.ncbi.nlm.nih.gov/genbank/, SRR5990720

https://www.ncbi.nlm.nih.gov/genbank/, SRR5990721.

## Author Contributions

GL, RO, SP, and RR conceived the study. SC, AB, and RR designed the experiments and wrote the manuscript with input from RO. SC, PM, CG, PD, LW, SM, WK, JL, and RR performed the experiments and/or generated data. SC, LW, PP, NS, KS, AB, and RR analyzed and interpreted the data. All authors contributed to the article and approved the submitted version.

### Conflict of Interest

The authors declare that the research was conducted in the absence of any commercial or financial relationships that could be construed as a potential conflict of interest.
