# Supplementary material for: An xa5 Resistance Gene-Breaking Indian Strain of the Rice Bacterial Blight Pathogen Xanthomonas oryzae pv. oryzae Is Nearly Identical to a Thai Strain
Source: Front Microbiol. 2020 Oct 21;11:579504. doi: 10.3389/fmicb.2020.579504 (PMC7610140; doi:10.3389/fmicb.2020.579504)
Supplement: Supplementary file 1 [file Data_Sheet_1.pdf]

Supplementary Material

An *xa5* resistance gene-breaking Indian strain of the rice bacterial blight pathogen *Xanthomonas oryzae* pv. oryzae is nearly identical to a Thai strain

Sara C. D. Carpenter, Prashant Mishra, Chandrika Ghoshal, Prasanta Dash, Li Wang, Samriti Midha, Gouri S. Laha, Jagjeet S. Lore, Wichai Kositratana, Nagendra K. Singh, Kuldeep Singh, Prabhu B. Patil, Ricardo Oliva, Sujin Patarapuwadol, Adam J Bogdanove*, Rhitu Rai*

*** Correspondence:** Adam J. Bogdanove (ajb7@cornell.edu) or Rhitu Rai (rhiturai@nrcpb.org)

1 Supplementary Tables

Supplementary Table S1: Primers used for qPCR

Primer name	Primer sequence	Source
18S Fwd	CTACGTCCCTGCCCTTTGTACA	(Jain et al., 2006)
18S Rev	ACACTTCACCGGACCATTCAA	(Jain et al., 2006)
SWEET11 Fwd	GACGACAGATTCTCGCTACTG	This study
SWEET11 Rev	TGACTGACTGACTGACTGACTGAC	This study
OsTFX1 Fwd	CAG CCATGCGAGAATATTTG	This study
OsTFX1 Rev	GGGCAGTTAGAATATTCCCG	This study
OsTFIIAγ1 Fwd	GCTGGAGACTAAAGAAGAGTAATTTGC	(Sugio et al., 2007)
OsTFIIAγ1 Rev	GAGCACACTCATGTCACACATCAC	(Sugio et al., 2007)

2 Supplementary Figures

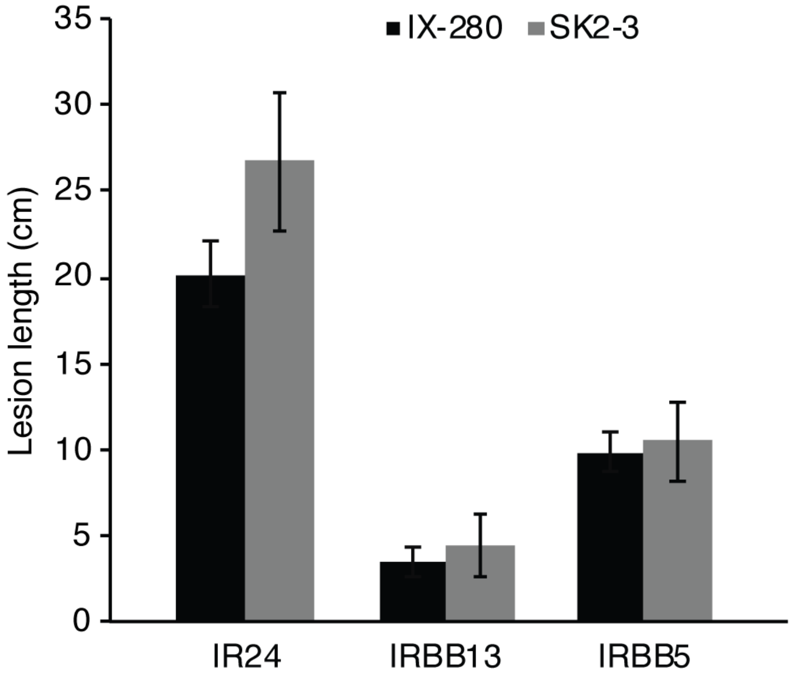

Supplementary Figure S1: Lesion lengths in near-isogenic lines IR24, IRBB13 and IRBB5 resulting from inoculation with IX-280 or SK2-3. Measurements were taken at 14 days after leaf clip inoculation using 45-day-old seedlings. Values are averages of 15 leaves each. Error bars denote standard deviation.

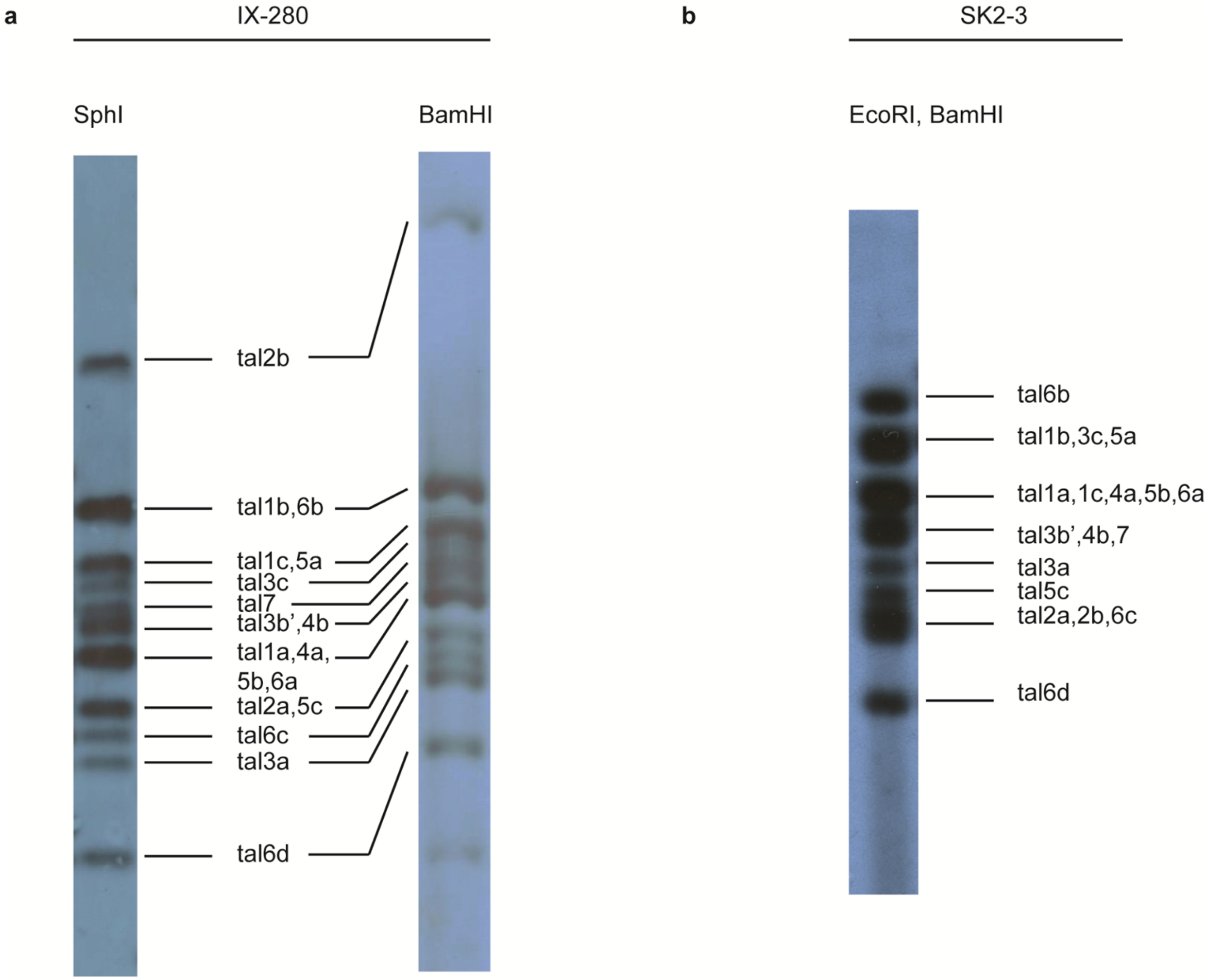

Supplementary Figure S2: Southern analysis of *tal* gene content in IX-280 and SK2-3. A. IX-280 genomic DNA digested with either *Sph*I or *Bam*HI and probed with *avrXa7*. B. SK2-3 digested with both *Eco*RI and *Bam*HI and probed with *avrXa7*.

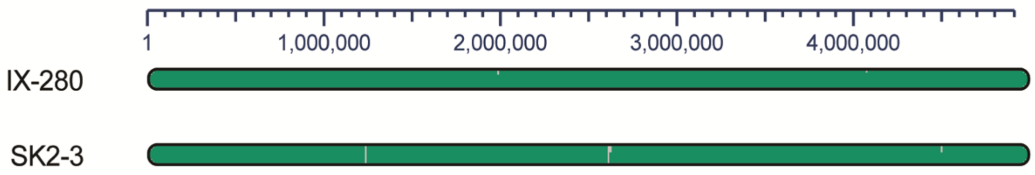

Supplementary Figure S3: Progressive Mauve alignment of IX-280 and SK2-3 chromosomes. Colinear blocks are shown in green, with grey indicating regions of sequence dissimilarity. The ruler indicates distance from the annotated origin in base pairs.

	1	2	3	4	5	6	7	8	9	10	11	12	13	14	15	16	17	18	19	20	21	22	23	24	25	26	27	In	% DNA Identity	PXO99A Ortholog	AnnoTALE Class	RVD Differences with PXO99A Ortholog
Tal1c	NN	HD	NI	**NG**	HD	NG	N*	HD	HD	NI	NG	NG	NI	HD	NG	NN	NG	NI	NI	NI	NI	N*	NS	N*				Both	99	Pthxo1	TalBX	NG4HG
Tal1b	NI	HG	NI	NN	NS	HD	NN	HD	HG	HD	NI	NI	NN	NI	HD	HD	HD	HG	NN	NN	HD	NS	NN	HD	N*	NS	N*	Both	100		TalAS	
Tal1a	NI	NG	NN	NG	NK	NG	NI	NN	NI	NN	NI	**NN**	**NS**	**NG**	**NS**	**NN**	**NI**	**N***	**NS**	**NG**								Both	99	Tal2a	TalAG	NN12HD, NS13N*, NG14NS, NS15N*, 16-20 absent
Tal2b†	NS	NG	NG	NG	NG	HD	HD	NN	NG	H*																		SK2-3	-		TalDW	
Tal2b†	NS	NG	NG	NG	NG	HD	HD	NN	NG	HD	NG	NG	HD	HD	HD	H*												IX-280	-		TalDW	
Tal2a†	NS	HD	NG	NG!	HG	NG	HD	HD	NG	HD	NN	HD	NG	HD	NI	NI	NI	N*										Both	99		TalDX	
Tal3c	NI	H*	NI	NN	NN	NN	NN	NN	HD	NI	HD	HG	HD	NI	N*	NS	NI	NI	HG	HD	NS	NS	NG					Both	100	PthXo6	TalAR	
Tal3b'	NI	HG	*ns*	*hg*	*hg*	*hd*	*ns*	*ng*	*hd*	*nn*	*ng*	*hg*	*ng*	*hd*	*hg*	*hd*	*hd*	*ni*	*nn*	*ng*								Both	100	Tal7b	TalAA	
Tal3a	NI	NS	HD	HG	NS	NN	HD	H*	NG	NN	NN	HD	HD	NG	HD	NG												Both	100	Tal5a	TalBA	
Tal4a	NI	N*	NI	NS	NN	NG	NN	NS	N*	NS	NN	NS	N*	NI	HG	HD	NI	HD	HD	NG								Both	100	Tal6a	TalAH	
Tal4b	NI	HG	NI	HG	NI	NI	NI	HD	NN	HD	NS	NG	SS	HD	NI	NI	NN	NI	NN	NI	NG							Both	99	Tal6b'	TalAN	
Tal5a	NN	HD	NS	NG	HD	NN	N*	NI	HD	NS	HD	NN	HD	NN	HD	NN	NN	NN	NN	NN	NN	NN	HD	NG				Both	99	Tal9e	TalAD	
Tal5b	NI	HG	NI	NI	NI	NN	HD	NS	NN	NS	NN	HD	NN	NI	HD	NN	**NI**	NG	**HD**	**NG**								Both	100	Tal7a/8a	TalAB	NI17NS, 19-20 absent
Tal5c	NI	NN	N*	NG	NS	NN	HD	N*	NN	NN	NI	NN	HD	NG	HD	HD	HD	NG										Both	100		TalAL	
Tal6a	HD	HD	HD	NG	N*	NN	HD	HD	N*	NI	NI	NN	HD	HI	ND	HD	NI	HD	NG	NG								Both	100	Tal9a	TalAP	
Tal6b	HD	HD	NN	NN	**NI**	NG	HD	**S***	HG	HD	NG	N*	**NG**	HD	HD	N*	**NI**	**NI**	NN	HD	HI	ND	HD	**NG**	NN	HG	N*	Both	100	Tal9b	TalAQ	NI5NG, S*8NS, NG13HD, NI17NN, NI18NI!, NG24HG
Tal6c	NI	NN	N*	NG	NS	NN	NN	NN	NI	NN	NI	**NG**	HD	HD	NI	**HG**	**N***											Both	99	AvrXa27	TalAO	NG12N*, HG16NG, N*17NG
Tal6d	NI	NN	NI	HG	HG	**HD**	**NG**	HD	HG	HD	HD	HD	NG															Both	100	Tal9d	TalAE	HD6NV, NG7HG
Tal7	NI	NG	NI	NI	N*	**HD**	HD	HD	N*	NI	NI	NI	NG	HD	HG	NN	NS	NN	HD	HD	NG	N*						Both	100	PthXo7	TalBM	HD6NN

Supplementary Figure S4: AnnoTALE classes and changes relative to PXO99A orthologues of IX-280/SK2-3 TAL effectors. RVDs in bold are different in PXO99A orthologs. A dagger indicates a truncTALE. The underlined RVD of Tal2a resides in a truncated (28 aa) repeat. Lower case italicized RVDs are untranslated following a frameshift. An asterisk indicates that the second amino acid in the RVD is absent, resulting in a 33 aa repeat. Percent DNA identity shown is that between TAL effectors of IX-280 and SK2-3. AnnoTALE classes in blue are new classes.

3 Supplementary References

Jain, M., Nijhawan, A., Tyagi, A.K., and Khurana, J.P. (2006). Validation of housekeeping genes as internal control for studying gene expression in rice by quantitative real-time PCR. *Biochem Biophys Res Commun* 345**,** 646-651.

Sugio, A., Yang, B., Zhu, T., and White, F.F. (2007). Two type III effector genes of *Xanthomonas oryzae* pv. oryzae control the induction of the host genes OsTFIIAgamma1 and OsTFX1 during bacterial blight of rice. *Proc Natl Acad Sci U S A* 104**,** 10720-10725.
